# Clinical Proteomics: From Biological Sample to Clinical Exploitation

**DOI:** 10.3390/proteomes5020010

**Published:** 2017-04-06

**Authors:** Edwin Lasonder

**Affiliations:** School of Biomedical and Healthcare Sciences, Plymouth University, Plymouth, Drake Circus, PL4 8AA, UK; edwin.lasonder@plymouth.ac.uk

Technological advances in mass spectrometry instrumentation and proteomics methodologies are moving the field of clinical proteomics towards the analysis of large numbers of patient samples in a reasonable time. The Special Issue ‘Clinical proteomics’ reviews the current proteomic work flow from biological sample preparation to clinical exploitation with samples collected from human body fluids, tissues or isolated cells, and highlights the trend towards integrated omics approaches for clinical usage.

Hernandez-Valladaras and colleagues [1] provide an overview of sample processing methods for acute myeloid leukemia cells (AML) harvested in bone marrow and peripheral blood from patients suffering from hematopoietic disease. AML biomarkers are promising targets for diagnostic and prognostic purposes enabling individual treatment regimens and identifying patients requiring bone marrow transplant. The review article compares the performance of urea-based lysis methods versus the filter-aided sample preparation (FASP) procedures and concludes that FASP-based procedures outperform standard in-solution digestion procedures and recommends to explore sample preparation methods for other cancer cells before analysing large patient cohorts. Peptide fractionation of AML protein digests is encouraged for obtaining in depth proteomes and phosphoproteomes by combining FASP with C18 STAGE tip fractionation steps and with IMAC as phosphopeptide enrichment step.

Sample processing and bioanalytical workflows dedicated to phosphoproteome analysis of traditional human biological fluids such as serum/plasma, urine, cerebrospinal fluid (CSF), saliva and broncoalveolar lavage fluid are discussed by Giorginanni and Beranova-Giorgianni [2], together with an overview in protein depletion methods for the detection of low abundant biomarkers. Multiple Affinity Removal System (MARS) is a popular immuno-affinity column for specific removal of abundant proteins, despite the possibility of depleting some off-target proteins. ProteoMiner protein enrichment technology is another depletion approach that allows for detection of equal protein amounts within a complex protein sample by employing a bead-based library of combinatorial peptide ligands to concentrate low abundance proteins to specific peptide ligands and dilute high abundance proteins by washing off excess protein after saturating specific peptide ligands. The authors review phosphoproteome applications for a variety of diseases (various cancers and neurological disorders) and conclude that clinical applications are largely unexplored and partly complicated by the plethora of bioanalytical workflows currently in use, and they foresee a move to simpler workflows relying on ‘single shot’ analyses with extended liquid chromatography LC gradients.

Sample handling procedures of non-traditional human samples such as ear wax, saliva, vitreous humor, aqueous humor, tears, nipple aspirate fluid, breast milk / colostrum, cervical-vaginal fluid, nasal secretions, bronco-alveolar lavage fluid, and stools for the utilization in quantitative proteomic studies for diagnostic and disease treatment purposes are reviewed by Licier et al. [3]. The article provides an excellent overview of various protein labelling methods for quantitative proteomics and recent clinical research work conducted with the non-traditional samples.

Trenchevska et al. [4] discuss a targeted sample processing approach for intact proteins isolated from human samples by Mass Spectrometric Immunoassay (MISA), the mass spectrometric equivalent of the Enzyme-Linked Immunosorbent Assay (ELISA). In this assay microcolumns are immobilised with antibodies to capture target proteins from complex biological samples that are detected by MALDI-TOF MS or ESI MS after rinsing the affinity pipette to remove non-specifically bounded proteins. Quantitative MISA experiments require co-immobilisation of an internal reference standard with an antibody towards the target protein that is exogenous to human body fluids, for example directed against protein derivatives (His-tagged proteins), or directed against homologues proteins from other animal species. Exogenous protein is spiked into samples as protein standard for absolute quantification. The authors focus on MISA applications of clinically significant proteoisoforms arising from alternative splicing, single nucleotide polymorphisms (SNPs) and post translational modifications. Some of the well-established disease biomarkers such as cystatin C, prostate-specific antigen and cardiac troponin exist in several forms in vivo. The potential of this method is demonstrated by published MISA experiments for more than 20 protein targets, where the authors highlight their own contributions showing detection of Serum amyloid A (SAA) protein isoforms from individuals expressing various polymorphic variants as distinct peaks in MALDI-TOF mass spectra.

Apolipoproteins have also been shown to exist in vivo in several proteoisoforms, which are involved in lipid metabolism, and are associated with cardiovascular diseases, type 2 diabetes and Alzheimer Disease (AD). Martins [5] argues that the observed variation in the detection of Apo E isoforms by clinical proteomics of various proteins in body fluids, originates from biological variation rather than from technical variation. Complementary technologies utilising genomics and lipidomics are therefore crucial for early stage diagnosis by revealing abnormalities in cholesterol levels and lipoprotein metabolism that are associated with cognitive and behavioral symptoms in later AD stages. Clinical genomics testing for Apo E is important for diagnosis avoiding errors by proteomics testing from the biological variation in amyloid beta and Apo E protein expression. Clinical lipidomics testing of plasma, tissue and CSF samples have provided reproducible results for the diagnosis of various neurological diseases including AD, which are also linked to chronic diseases. These complementary diagnostic tests suggest that nuclear receptors crucially determine the links between insulin resistance, chronic disease and AD, such as the downregulation of the calorie-sensitive anti-aging nuclear receptor Sirt1 in early stage of AD. The link between cholesterol and developing AD opens the door for treatment with nutritional diets maintaining cholesterol homeostasis. Nutritional proteomics research showed that AD plasma biomarkers are regulated by low fat diets through activation of Sirt1 preventingamyloid beta aggregation and induced inflammation.

The notion that detailed insights in cellular homeostasis and disease progression are generated from studies applying clinical proteomics with complementary omics technologies inspired Bosman for outlining the current perspective of red blood cell (RBC) homeostasis by integrating proteome with metabolome data [6]. Comparative proteomics studies have generated erythrocyte protein inventories and provided new insights in mechanisms controlling red blood cell morphology under pathological conditions. Protein kinase activities and signalling networks regulate pathology-associated changes in phosphorylation status of erythrocyte cytoskeleton proteins, such as band 3, and RBC membrane structure. Proteomics of the erythrocyte cytosolic fraction identified not only metabolic enzymes involved in glycolysis and pentose phosphate pathway but also protein-repair enzymes. These enzymes are thought to be assembled in multiprotein complexes regulating oxygen transport, metabolism, anti-oxidant activity and protein breakdown. Metabolomics data suggests that CO_2_ concentrations are also associated with the multiprotein complexes besides oxygen. The combination of proteome and metabolome data alludes to a mechanism with central roles for molecular interactions at the red cell surface regulating cell shape, deformability, cell survival, and metabolism with oxygen and CO_2_ transport. Support for this proposed mechanism comes from observations that glycolysis and the pentose phosphate pathway are regulated by oxygen-driven interactions of key enzymes with the membrane, and that binding between cytoskeleton and membrane is controlled by oxygen-mediated interaction between band 3 and ankyrin. Recent studies further establish relations between metabolic changes and erythrocyte pathologies, which are likely caused by changes in membrane-associated protein complexes.

The holistic view of the utilization of complementary mass spectrometry-based technologies in personalized proteomics for clinical diagnosis and treatment is seen by Duarte and Spencer [7] as the future for personalized medicine. Genomics information alone is insufficient for diagnosing multifactorial diseases such as AD by the inherent inability in gathering all information that predict physiological states of patients, such as protein expression patterns, protein-protein interactions, PTMs and metabolites. Current limitations for personalized proteomics in clinical practice are technological complexity of the analyses involved and lack of standardisation for sample processing procedures [1,2,3,4] and mass spectrometry methodology [1,2,3,7]. Furthermore, translation of proteomics-based biomarker discovery research into the clinic has been limited by failures in the validation phase in testing large patient cohorts. More integration of biomarker discovery phase research with clinical studies will lead to the implementation of validated biomarker assays in clinical practice and move clinical proteomics out of its infancy.
